# On‐ versus off‐pump CABG in octogenarians: A propensity‐matched analysis from the UK National Database

**DOI:** 10.1111/jocs.17068

**Published:** 2022-11-02

**Authors:** Jeremy Chan, Arnaldo Dimagli, Daniel P. Fudulu, Tim Dong, Ester Mikova, Gianni D. Angelini

**Affiliations:** ^1^ Bristol Heart Institute University of Bristol Bristol UK

**Keywords:** Coronary artery bypass grafting, Off Pump, Octogenarians

## Abstract

**Introduction:**

Coronary artery bypass grafting (CABG) remains a good revascularization strategy in octogenarians with excellent clinical outcomes and quality of life postoperatively. However, the benefits of off‐pump over on‐pump CABG in the elderly population are still controversial. We investigated this issue in the UK National Audit database.

**Method:**

We retrospectively analyzed all octogenarians undergoing nonemergency, isolated CABG from 1996 to 2019. Propensity score matching (PSM) was conducted to adjust for imbalance in the baseline characteristics between the off‐pump and on‐pump groups. Primary outcome was in‐hospital mortality and postoperative cerebrovascular accidents. Secondary outcomes were bleeding requiring reoperation, deep sternal wound infection, and postoperative dialysis.

**Result:**

A total of 6436 patients were included for analysis. No differences were observed between off‐ and on‐pump group in‐hospital mortality (4% vs. 3.8%, *p* = .89), return to theater rate (5.4% vs. 6.2%, *p* = .16) and incidence of deep sternal wound infection (1.1% vs. 1.6%, *p* = .34). However, octogenarian undergoing off‐pump CABG were less likely to experience postoperative transient ischemic attack (TIA)/stroke (1.4% vs. 2.3%, *p* = .004) but more likely to require renal dialysis (4.8% vs. 3.5%, *p* = .03).

**Conclusion:**

The data show similar in‐hospital mortality in octogenarians regardless of the revascularization technique used. Off‐pump when compared with on‐pump CABG is associated with a lower incidence in postoperative neurological events but a higher need for renal dialysis.

**Abbreviations**
AKI: Acute kidney injury
CABG: Coronary artery bypass grafting
CVA: Cerebrovascular accidents
MAG: Multi‐arterial grafting
NACSA: National Adult Cardiac Surgery Audit
ONCABG: On‐pump CABG
OPCABG: Off‐pump CABG
PS: Propensity score
PSM: Propensity score matching
SMD: Standardised mean difference

## 1 INTRODUCTION

There is growing proportion of octogenarian patients being offered coronary artery bypass grafting (CABG) surgery as the average life expectancy continues to rise in the developed world.^1^, ^2^ The number has increased almost threefold to 7.2% of all CABG performed in the United Kingdom from 2002 to 2016.^3^ Also, these patients are usually more comorbid than their younger counterpart and therefore, although CABG remains a good option, it is burdened with significant morbidity and mortality. It is, therefore, conceivable that avoiding exposure of these patients to the inflammatory stress of cardiopulmonary bypass and cardioplegic arrest may play a relevant role in improving their outcomes.

In this study, we performed a retrospective analysis of prospectively collected data from the UK National Adult Cardiac Surgery to evaluate short‐term outcomes between on‐pump and off‐pump CABG in Octogenarians.

## 2 METHOD

The study was part of a research project approved by the Health Research Authority (HRA) and Health and Care Research Wales. As the study included retrospective interrogation of the NICOR database the need for individual patient consent was waived off (HCRW) (IRAS ID: 278171) in accordance with the research guidance. The study was performed in accordance with the ethical standards as laid down in the 1964 Declaration of Helsinki and its later amendments.

**Figure 1:**
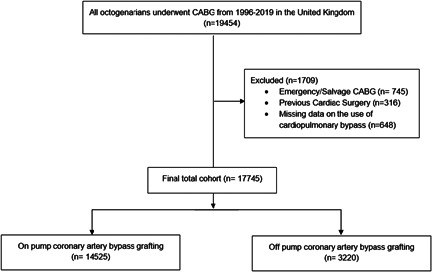
The CONSORT diagram in the study patient cohort applying exclusion criteria

All patients undergoing elective or urgent isolated CABG from 1996 to 2019 were extracted from the National Adult Cardiac Surgery Audit (NACSA) database. The NACSA database prospectively collects data on all major heart operations carried out on National Health Service patients in the United Kingdom since April 1996. The definitions of database variables used, and a description of the database were previously described.^4^ Patients were divided into two groups: (a) on‐pump CABG (ONCABG) and (b) off‐pump CABG (OPCABG). Patients who underwent emergency or salvage CABG, nonisolated CABG, underwent previous cardiac surgery and were less than 80 years old were excluded from the study. Patients with missing data regarding the use of cardiopulmonary bypass (*n* = 648) were also excluded from the analysis. A consort style diagram showing the study patient cohort applying inclusion and exclusion criteria is shown in Figure 1.

### 2.1 Study endpoints

The primary outcome is defined as in‐hospital mortality and postoperative cerebrovascular accidents (CVA). CVA were defined as transient ischemic attack or the occurrence of permanent stroke, diagnosed clinically and radiologically during the index hospitalization. Secondary outcomes were re‐exploration for bleeding, deep sternal wound infection, and postoperative dialysis

### 2.2 Statistical analysis

Continuous variables are presented as mean (SD) or median (IQR) and were compared using student's *t* test or Wilcoxon rank‐sum test, as appropriate. Categorical variables are presented as numbers and frequencies and were compared using *χ* ^2^ exact test. Missing continuous variables data (Creatinine [*n* = 9795], body mass index [BMI] [*n* = 954] and left ventricular ejection fraction [LVEF] [*n* = 11,511]) were imputed with the mean value in the data after the application of exclusion criteria listed above, while categorical variable (Gender [*n* = 5]) was imputed with the mode.

To account for potential imbalance in baseline risk, a propensity score (PS) was calculated for each patient on the basis of a nonparsimonious logistic regression model. All variables included in the model were listed in Table 1. A nearest neighbor propensity score matching (PSM) without replacement and with a caliper width of 0.2 standard deviations of the logit of the PS was performed. After PSM, standardized mean difference (SMD) was used to assess the balance of the covariates between the ONCABG and OPCABG groups. A value higher than 0.10 was considered to indicate the presence of residual imbalance among variables. The density of PS distribution in the control and treated groups before and after PSM is shown in the Histogram (Supporting Information:  Figure 1). A generalized, linear model was used to evaluate the association between outcomes and ONCABG versus OPCABG. Results are demonstrated as odds ratio (OR) and 95% confidence interval (95% CI). In all the analyses, ONCABG was used as the reference group. R (Version 4.1.1) and R Studio (Version 1.4.1103, RStudio, PBC) were used to perform the statistical analysis. A *p* value of less than .05 is deemed statistically significant.

**Table 1:** The preoperative characteristics of the two groups on‐pump and off‐pump: All patients and propensity matched

Preoperative characteristics	All patients			Propensity matched			
	Off‐pump (*n* = 3220)	On pump (*n* = 14,525)	*p* value	Off‐pump (*n* = 3218)	On pump (*n* = 3218)	SMD	*p* value
Age (Year)	82.65 (2.16)	82.55 (2.09)	.050	82.65 (2.17)	82.62 (2.12)	0.0158	.89
Gender (Male)	2340/3220 (73%)	10,681/14,525 (74%)	.32	2338/3218 (73%)	2337/3218 (73%)	0.0007	.98
BMI	26.50 (3.72)	26.79 (4.03)	<.001	26.50 (3.72)	26.73 (3.81)	−0.0553	.01
Urgency			.003				.12
Elective	1718/3220 (53%)	7326/14525 (50%)		278/3218 (8.6%)	316/3218 (9.8%)	−0.0366	
Urgent	1502/3220 (47%)	7119/14525 (50%)		278/3218 (8.6%)	254/3218 (7.9%)	0.0274	
CCS grade			<.001	1023/3218 (32%)	1025/3218 (32%)	−0.0013	
0	278/3220 (8.6%)	1718/14,525 (12%)		1114/3218 (35%)	1051/3218 (33%)	0.0419	
1	279/3220 (8.7%)	1171/14,525 (8.1%)		525/3218 (16%)	572/3218 (18%)	−0.0393	
2	1023/3220 (32%)	4551/14,525 (31%)					.99
3	1115/3220 (35%)	4684/14,525 (32%)		842/3218 (26%)	846/3218 (26%)	−0.0028	
4	525/3220 (16%)	2401/14,525 (17%)		1412/3218 (44%)	1414/3218 (44%)	−0.0012	
NYHA status			.032	829/3218 (26%)	824/3218 (26%)	0.0036	
1	843/3220 (26%)	3813/14,525 (26%)		135/3218 (4.2%)	134/3218 (4.2%)	0.0017	
2	1412/3220 (44%)	6705/14,525 (46%)		271/3218 (8.4%)	244/3218 (7.6%)	0.0318	.21
3	830/3220 (26%)	3475/14,525 (24%)		2/3218 (<0.1%)	4/3218 (0.1%)	−0.0216	.69
4	135/3220 (4.2%)	532/14,525 (3.7%)					.56
Preop AF	272/3220 (8.4%)	1094/14,525 (7.5%)	.078	1551/3218 (48%)	1508/3218 (47%)	0.0268	
LMS	2/3220 (<0.1%)	12/14,525 (<0.1%)	.99	1317/3218 (41%)	1351/3218 (42%)	−0.0214	
Previous MI			.13	350/3218 (11%)	359/3218 (11%)	−0.0094	
0	1551/3220 (48%)	6924/14,525 (48%)					.62
1	1319/3220 (41%)	6165/14,525 (42%)		2954/3218 (92%)	2960/3218 (92%)	−0.0069	
2 or more	350/3220 (11%)	1436/14,525 (9.9%)		13/3218 (0.4%)	11/3218 (0.3%)	0.0129	
Previous PCI			.042	29/3218 (0.9%)	38/3218 (1.2%)	−0.0254	
0	2954/3220 (92%)	13,366/14,525 (92%)		222/3218 (6.9%)	209/3218 (6.5%)	0.0164	
1	15/3220 (0.5%)	34/14,525 (0.2%)		50.52 (5.76)	50.45 (6.33)	0.0117	.42
2	29/3220 (0.9%)	179/14,525 (1.2%)					.89
3+	222/3220 (6.9%)	946/14,525 (6.5%)		2567/3218 (80%)	2558/3218 (79%)	0.0068	
LVEF	50.52 (5.76)	50.47 (6.48)	.33	119/3218 (3.7%)	130/3,218 (4.0%)	−0.0162	
Diabetes			.054	419/3218 (13%)	413/3218 (13%)	0.0056	
No	2569/3220 (80%)	11,402/14,525 (78%)		113/3218 (3.5%)	117/3218 (3.6%)	−0.0064	
Diet control	119/3220 (3.7%)	680/14,525 (4.7%)					.79
Drug control	419/3220 (13%)	1868/14,525 (13%)		1245/3218 (39%)	1218/3218 (38%)	0.0172	
Insulin	113/3220 (3.5%)	575/14,525 (4.0%)		1885/3218 (59%)	1911/3218 (59%)	−0.0164	
Smoking status			.48	88/3218 (2.7%)	89/3218 (2.8%)	−0.0020	
Non smoker	1246/3220 (39%)	5760/14,525 (40%)		398/3218 (12%)	382/3218 (12%)	0.0156	.54
Ex‐smoker	1886/3220 (59%)	8404/14,525 (58%)		68/3218 (2.1%)	89/3218 (2.8%)	−0.0430	.09
Current smoker	88/3220 (2.7%)	361/14,525 (2.5%)		552/3218 (17%)	530/3218 (16%)	0.0158	.89
Pul Disease	398/3220 (12%)	1667/14,525 (11%)	.16	82.65 (2.17)	82.62 (2.12)	0.0007	.98
NeuroDys	68/3220 (2.1%)	343/14,525 (2.4%)	.39	2338/3218 (73%)	2337/3218 (73%)	−0.0553	.01
PVD	553/3220 (17%)	2323/14,525 (16%)	.10	26.50 (3.72)	26.73 (3.81)	0.0187	.12

*Note*: Data are expressed as mean ±SMD.

Abbreviations: AF, atrial fibrillation; BMI, body mass index; CCS, Canadian Cardiovascular Society; ES2, Euro Score II; LVEF, left ventricular ejection fraction; LMS, left main stem disease; MI, myocardial infraction; NYHA, New York Heart Association; NeuroDys, neurological dysfunction; PCI, percutaneous coronary intervention; Pul Disease, pulmonary disease; PVD, peripheral vascular disease.

## 3 RESULTS

**Figure 2:**
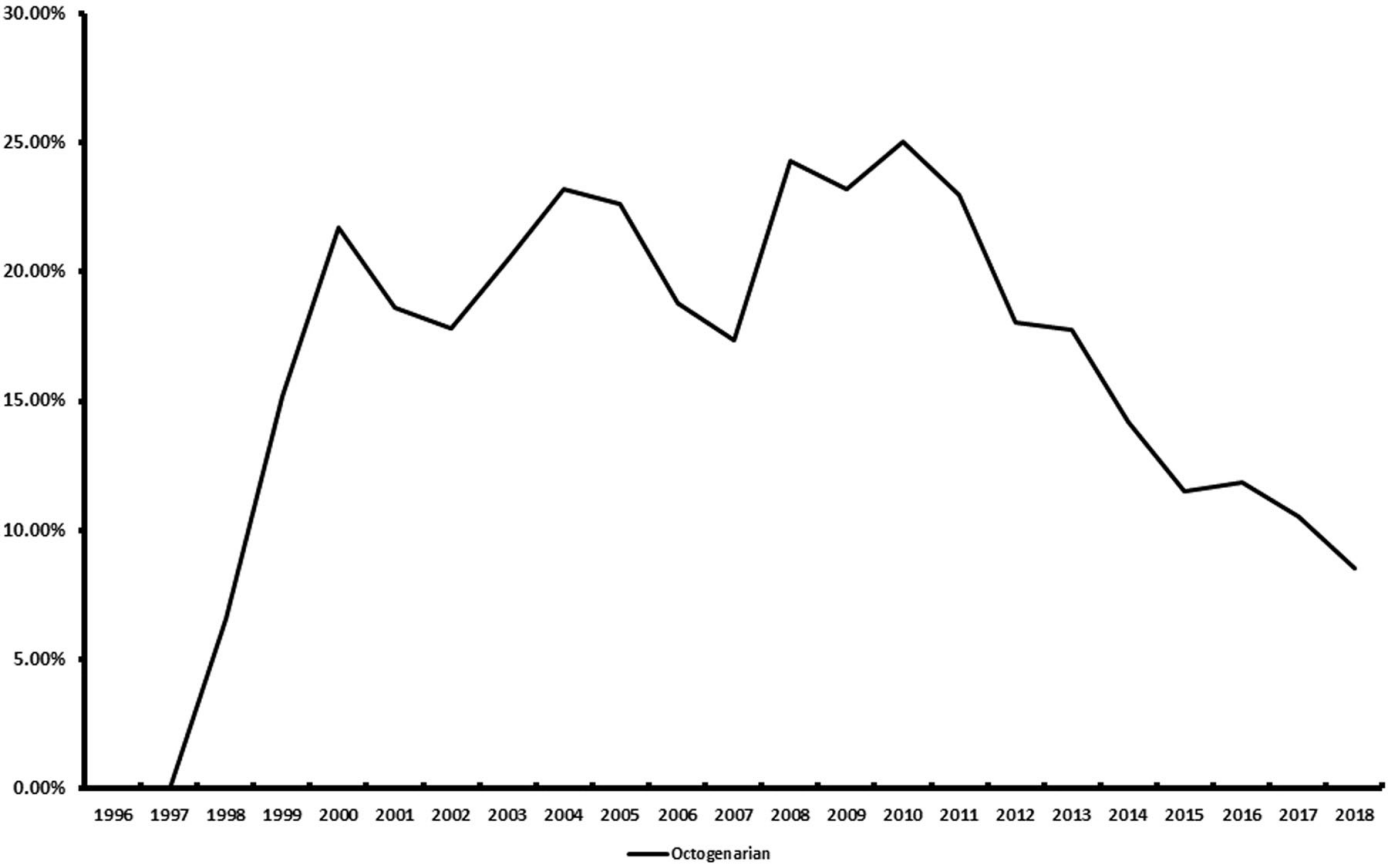
The trends of off‐pump coronary artery bypass grafting (CABG) performed in octogenarians over the studied period

A total of 17,745 octogenarians underwent CABG over the study period, of which 14,525 (81.85%) underwent ONCABG. The mean EuroScore II for OPCABG and ONCABG was 2.66 (2.62) and 2.86 (2.57), respectively. The proportions of CABG performed in octogenarians using off‐pump technique peaked in 2010 (25.02%) to 2018 (8.55%). Figure 2 and Supporting Information: Table 1 showed the trends of OPCABG performed in octogenarians over the study period.

### 3.1 Preoperative characteristics

After PSM, 6436 patients were included in the analysis. Preoperative patient demographics and comorbidities before and after PSM are presented in Table 1. Younger patients (odds ratio [OR]: 0.98, 95% confidence interval [CI]: 0.96–0.99, *p* = .01) were more likely to undergo OPCABG while patients with increased BMI (OR: 1.02, 95% CI: 1.01–1.03, *p* < .001) and nonelective procedures (OR: 1.16, 95% CI: 1.06–1.26, *p* < .001) were more likely to undergo ONCABG. Table 2 shows the predictors for OPCABG based on baseline characteristics.

**Table 2:** The predictors for off‐pump coronary artery bypass grafting based on baseline characteristics

Patient characteristics	OR (95% CI)	*p* value
Age (year)	0.98 (0.96–0.99)	.01
Gender (male sex)	0.93 (0.85–1.02)	.11
Smoking	0.89 (0.69–1.13)	.33
Pulmonary disease	0.93 (0.83–1.05)	.23
Neurological dysfunction	1.13 (0.87–1.47)	.36
Peripheral vascular disease	0.93 (0.84–1.04)	.19
Diabetes	1.12 (0.91–1.38)	.28
LV ejection fraction	1.00 (0.99–1.01)	.66
Body mass index	1.02 (1.01–1.03)	<.001
Preoperative AF	0.88 (0.77–1.02)	.08
Urgent operation	1.16 (1.06–1.26)	<.001
Creatinine Level	1.00 (0.99–1.01)	.082
Left main stem disease	1.25 (0.28–5.62)	.77

Abbreviations: AF, atrial fibrillation; BMI, body mass index; CI, confidence interval; LV, left ventricle; OR, odds ratio.

### 3.2 Intraoperative characteristics

The mean cardiopulmonary bypass and cross‐clamp time for the ONCABG group were 84.0 (39.42) and 48.1 (24.4) minutes, respectively. The mean number of grafts performed were three in both groups. Octogenarians who underwent OPCABG were more likely to receive >1 arterial graft (18.9% vs. 5.6%, *p* < .001).

### 3.3 Postoperative characteristics

There were no differences between OPCABG and ONCABG in in‐hospital mortality (4% vs. 3.6%, OR: 0.98, 95% CI: 0.76–1.27, *p* = .89), return to theater for bleeding (5.4% vs. 6.2%, OR: 1.17, 95% CI: 0.94–1.46, *p* = .16), and incidence of deep sternal wound infection (1.1% vs. 1.6%, OR: 1.49, 95% CI: 0.66–3.66, *p* = .34). However, octogenarians undergoing OPCABG were less likely to develop postoperative TIA/stroke (1.4% vs. 2.3%, OR: 1.83, 95% CI: 1.21–2.76, *p* = .004) but more likely to require renal dialysis (4.8% vs. 3.5%, OR: 0.74, 95% CI: 0.57–0.96, *p* = .03). Table 3 shows the intra‐ and postoperative outcome between on‐pump and off‐pump groups.

**Table 3:** shows the intra‐ and postoperative outcome between on‐pump and off‐pump groups after propensity score matching

Characteristics	All patients	Propensity matched				
	Off‐pump (*n* = 3220)	On pump (*n* = 14525)	*p* value	Off pump (*n* = 3218)	On pump (*n* = 3218)	*p* value
CPB (minute)	N/A	83.21 (33.67)	N/A	N/A	84.00 (39.42)	N/A
XClamp (minute)	N/A	47.95 (21.62)	N/A	N/A	48.09 (21.41)	N/A
MAG	604 (19%)	784 (5.4%)	<.001	602 (19%)	180 (5.6%)	<.001
Mortality	128 (4%)	616 (4.3%)	.55	128 (4.0%)	123 (3.8%)	.69
Return to theater	165 (5.4%)	852 (6.6%)	.018	165 (5.4%)	177 (6.2%)	.20
Postop stroke			.023			.006
TIA	18 (0.7%)	101 (0.8%)		18 (0.7%)	23 (0.8%)	
CVA	18 (0.7%)	156 (1.2%)		18 (0.7%)	43 (1.5%)	
Postop dialysis	133 (4.8%)	478 (3.8%)	.019	133 (4.8%)	98 (3.5%)	.019
Postop deep sternal wound infection	9 (1%)	75 (1.5%)	.27	9(1.1%)	17 (1.6%)	.31

*Note*: Data are expressed as mean ±SD.

Abbreviations: CI, confidence interval; CPB, cardiopulmonary bypass; CVA, cerebral vascular accident; MAG, multiple arterial grafts; N/A, not applicable; OR, odds ratio; Postop, postoperative; TIA, transient ischemic attack; XClamp, cross clamp.

## 4 DISCUSSION

The aging population in developed countries continue to rise and the need for coronary revascularisation for octogenarians have increased. In the past, age was a criterion itself upon which surgical intervention was denied. With the advancement in peri‐operative management, more octogenarians are now offered CABG for coronary revascularisation. Indeed, the number of octogenarians consisted of 7.2% of all CABG performed in the United Kingdom from 2002 to 2016 and is continuing to rise.^1^ This further raises the importance to identify the best revascularisation strategy in this group. OPCABG avoids the use of cardiopulmonary bypass and cardioplegic arrest and thus reduces the potential complications from aortic manipulation and the sequelae related to the induced inflammatory status. Reduction in the incidence of stroke postoperatively has been widely reported in both the general population and the high‐risk groups for patients undergoing OPCABG.^5^, ^6^ However, OPCABG is also more technically demanding than ONCAB and controversy remains regarding the potential for incomplete revascularisation, which itself is associated with poorer prognosis.^7^, ^8^ However, when performed by experienced surgeons, long‐term health outcome were similar between the two groups.^9^

The evidence with regard to this topic remains contradictory. Neither technique has solid evidence supporting its benefits on reduction in in‐hospital mortality. Studies comparing OPCAB with ONCAB performed in the early 2000–2010s showed a reduction a in postoperative complications and in‐hospital mortality in octogenarians and the general population.^10^, ^11^, ^12^ A recent meta‐analysis reported lower in‐hospital mortality in octogenarians underwent OPCAB, in addition to a reduction in the incidence of postoperative stroke and length of hospital stay.^13^ However, such superiority faded off when comparing the early and midterm survival rates between the two‐revascularisation technique.^7^, ^10^, ^12^ OPCAB seems to be associated with an increased incidence of incomplete revascularisation, which is independently associated with a poor outcome in the octogenarian cohort.^7^ Two of the three recent articles using national/regional registry also failed to show a significant difference in in‐hospital survival regardless of revascularisation technique.^14^, ^15^, ^16^

Several cohort studies have examined the long‐term survival benefits of OPCABG in octogenarians. Chikwe et al. reported a long‐term (10 years) survival benefit in the OPCABG group, performed by experienced (>100 cases) surgeons,^17^ while Knapik et al., reported the long‐term (12 years) survival benefits in the OPCABG cohort using the Polish KROK registry.^14^

Currently, the GOPCABE trial is the only randomized control trial compared ONCAB versus OPCAB in the elderly (>75 years old) population.^7^ No differences in 5‐year survival rate, myocardial infarction and repeat revascularisation rate between the two groups were observed. Instead, the only factor associated with a poor 5‐year survival rate was incomplete revascularization. While most studies focus on biological age, the ongoing FRAGILE study focuses on clinical frailty and aims to compare the effect of on‐ and off‐pump technique in frail patients (≥2 criteria of frailty by Fried Frailty Criteria) who undergo CABG.^18^, ^19^

One major benefit of OPCABG is minimizing the need for/reduce the times of manipulation of the ascending aorta by avoiding cannulation, potentially leading to a reduction in postoperative neurological events. Our data showed on‐pump CABG was associated with an increased risk of postoperative neurological events (TIA/CVA) in octogenarians. Similar findings were reported in three other large studies examining national registries.^13^, ^15^, ^20^ The use of OPCABG allows surgeons to minimize or completely avoid (anaortic) the manipulation of the aorta.^21^ This is particularly beneficial in patients with the severe atherosclerotic aorta.

Acute kidney injury (AKI) and the need for dialysis is a recognized complication of CABG. The use of cardiopulmonary bypass induced a generalized systemic inflammatory response, nonpulsatile flow, haemodilution, renal hypoperfusion that could lead to a 5%–20% increases in serum creatinine.^22^, ^23^, ^24^, ^25^ The incidence of postoperative AKI was higher in patients underwent ONCABG when compared with OPCABG.^26^, ^27^, ^28^ However, such effect did not translate into the increased need for dialysis.^27^, ^29^ Similar results were also observed in the octogenarian cohort.^7^, ^14^, ^15^, ^24^, ^30^ This is different from our study, which demonstrated an increased incidence of postoperative renal dialysis in the OPCABG group.

Our data also demonstrate 18.9% of octogenarians who underwent OPCABG received more than 1 arterial graft. Limited data showed an intermediate (1–5 years) and midterm (>5 years) survival benefits of multiarterial grafting over single arterial graft in elderly patients.^31^, ^32^ The use of ESC/EACTS guidelines recommend the use of multiarterial grafting in patients with a life expectancy >5 years.^33^ Octogenarians who underwent surgical revascularisation would likely fit in this category and should be considered for multiarterial grafting in selected cases.

## 5 LIMITATIONS

There are several limitations to our study. The NACSA database heavily relies on health‐care professionals' input and some data were missing in some parts of the analysis. This is particularly apparent in the postoperative outcome and some of the nonmandatory options in the database. A 3.52% of data on cardiopulmonary bypass use was missing and required to be excluded from the analysis. Despite the application of PSM, residual bias may be present in the analysis since the propensity‐matched model can account only for measured confounders and not for the unmeasured confounders (e.g., frailty). The absence of conversion from off‐pump to on‐pump, long‐term follow‐up data showing survival rate, the need for revascularisation and major adverse cardiac events rate is another limitation. Nevertheless, we believe our study is an important topic in surgical practice worldwide.

## 6 CONCLUSION

Our data showed similar in‐hospital mortality in octogenarians regardless of the revascularisation technique used. Off‐pump when compared with on‐pump CABG was associated with a lower incidence in postoperative neurological events but a higher need for renal dialysis.

## AUTHOR CONTRIBUTIONS

**Jeremy Chan**: Conceptualization; data curation; formal analysis; methodology; writing–original draft; writing–review & editing. **Arnaldo Dimagli**: Data curation; formal analysis; methodology; writing–original draft; writing–review & editing. **Daniel P. Fudulu**: Conceptualization; writing–review & editing. **Gianni D. Angelini**: Conceptualization; supervision; methodology; writing–review & editing.

## CONFLICT OF INTEREST

The authors declare no conflict of interest.

## Supporting information

Supplementary information.
