# Supplementary material for: On‐ versus off‐pump CABG in octogenarians: A propensity‐matched analysis from the UK National Database
Source: J Card Surg. 2022 Nov 2;37(12):4705–12. doi: 10.1111/jocs.17068 (PMC10092246; doi:10.1111/jocs.17068)
Supplement: Supplementary file 1 — Supplementary information. [file JOCS-37-4705-s001.docx]

| Year | Off pump | % | On Pump | % | Total |
| --- | --- | --- | --- | --- | --- |
| 1996 | 0 | 0.00% | 16 | 100.00% | 16 |
| 1997 | 0 | 0.00% | 44 | 100.00% | 44 |
| 1998 | 4 | 6.56% | 57 | 93.44% | 61 |
| 1999 | 17 | 15.18% | 95 | 84.82% | 112 |
| 2000 | 38 | 21.71% | 137 | 78.29% | 175 |
| 2001 | 62 | 18.62% | 271 | 81.38% | 333 |
| 2002 | 87 | 17.83% | 401 | 82.17% | 488 |
| 2003 | 151 | 20.43% | 588 | 79.57% | 739 |
| 2004 | 183 | 23.22% | 605 | 76.78% | 788 |
| 2005 | 174 | 22.63% | 595 | 77.37% | 769 |
| 2006 | 175 | 18.82% | 755 | 81.18% | 930 |
| 2007 | 184 | 17.34% | 877 | 82.66% | 1061 |
| 2008 | 287 | 24.28% | 895 | 75.72% | 1182 |
| 2009 | 284 | 23.22% | 939 | 76.78% | 1223 |
| 2010 | 323 | 25.02% | 968 | 74.98% | 1291 |
| 2011 | 279 | 22.96% | 936 | 77.04% | 1215 |
| 2012 | 216 | 18.06% | 980 | 81.94% | 1196 |
| 2013 | 194 | 17.73% | 900 | 82.27% | 1094 |
| 2014 | 142 | 14.20% | 858 | 85.80% | 1000 |
| 2015 | 118 | 11.53% | 905 | 88.47% | 1023 |
| 2016 | 119 | 11.86% | 884 | 88.14% | 1003 |
| 2017 | 105 | 10.55% | 890 | 89.45% | 995 |
| 2018 | 70 | 8.55% | 749 | 91.45% | 819 |

Table 1 showed the number of octogenarians undergoing on and off pump coronary artery bypass grafting from 1996-2018.


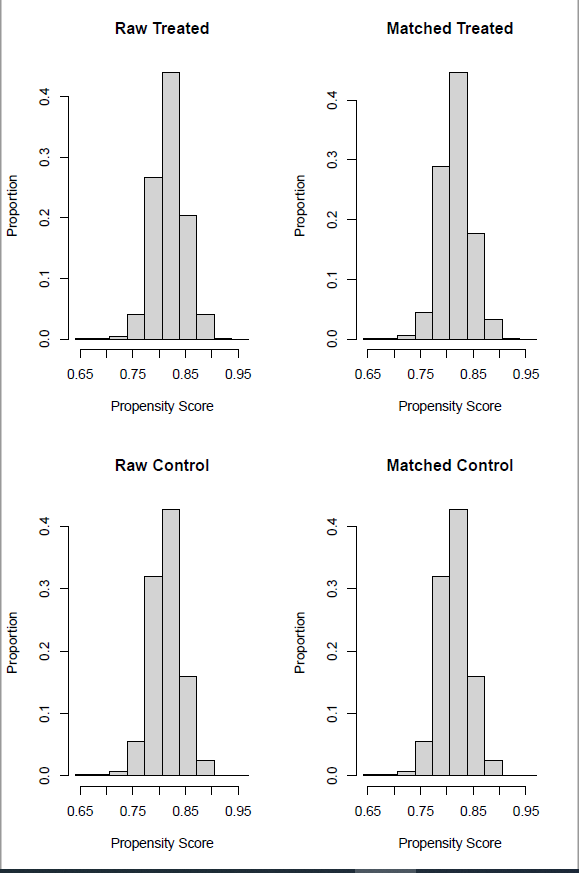


Figure 1 showed the density of propensity score distribution in the control and treated groups before and after propensity score matching
